# Simplified Medication Adherence Questionnaire (SMAQ) for People Living With HIV in a National Hospital in Mexico: Instrument Validation Study

**DOI:** 10.2196/59562

**Published:** 2025-01-07

**Authors:** Luis Eduardo Del Moral Trinidad, Luz Alicia González Hernández, Jaime Federico Andrade Villanueva, Pedro Martínez-Ayala, Adriana Valle Rodríguez, Vida Veronica Ruíz Herrera, José Adán Vizcaíno Résendiz, Melva Guadalupe Herrera Godina, Sergio Dominguez-Lara

**Affiliations:** 1 Department of Public Health Sciences University of Guadalajara Guadalajara Mexico; 2 HIV Unit Hospital Civil de Guadalajara Guadalajara Mexico; 3 Institute of Biomedical Sciences Research University of Guadalajara Guadalajara Mexico; 4 Research Institute Facultad de Ciencias de la Comunicación, Turismo y Psicología University of San Martín de Porres Lima Peru

**Keywords:** treatment adherence, HIV, Mexico, validation, Spanish, Hispanic, cross sectional, surveys, questionnaires, scales, adherence, viral load, sexually transmitted infection, STI, drugs, pharmacotherapy, medication, simplified medication adherence questionnaire, SMAQ

## Abstract

**Background:**

Adherence to antiretroviral therapy is a critical component in achieving viral suppression in people living with HIV in addition to increasing overall quality of life. Several indirect methods have been used to measure adherence including the Simplified Medication Adherence Questionnaire (SMAQ).

**Objective:**

The objective of this study is to evaluate the reliability and validity of the SMAQ in men living with HIV/AIDS attending a Mexican national hospital.

**Methods:**

A cross-sectional analytical design study was carried out in a Mexican National Hospital in Jalisco, including men aged >18 years with at least 3 months of antiretroviral treatment, excluding those with cognitive difficulties in answering the survey. A minimum sample size was calculated to detect the contribution of the variables within the model. The analysis included descriptive tests, confirmatory factor analysis, reliability and validity assessment, correlation between adherence and viral load, and association between viral load and adherence.

**Results:**

The final analysis included a total of 260 patients with a mean age of 43 (SD 12) years and an average of 8.97 (SD 6.33) years on antiretroviral treatment. The SMAQ showed sufficient structural validity (comparative fit index=1, root-mean-square error of approximation=0, 90% CI 0-0.085) with satisfactory factor loadings on most questions except item 2 (Do you always take your medication at the prescribed time?). The reliability of the scale is acceptable (Cronbach α=0.702, ω=0.718). Adherence correlated with viral load significantly but not with recent TCD4 lymphocyte levels. Patients classified as adherent were three times more likely to be undetectable than nonadherent patients (odds ratio 3.31, 95% CI 1.13-9.64, *P*=.04).

**Conclusions:**

The SMAQ represents an adequate tool to assess adherence in men living with HIV in the Mexican context, this will contribute to this study and compression of adherence to establish future intervention programs.

## Introduction

According to the Joint United Nations Program on HIV/AIDS, 39 million people were living with HIV by 2022, of which only 76.4% were receiving antiretroviral treatment (ART). In Mexico, by 2022, there were 270,000 cases registered with the Ministry of Health, 80% (n=270,000) of which were men; in addition, Jalisco ranks 4th in prevalence of people living with HIV with a record of 7134 patients on ART and 78% of this population has achieved viral suppression through such treatment [1,2].

The World Health Organization defines adherence to treatment as an individual’s behavior regarding medications, diet, and lifestyle changes that correspond to the recommendations provided by a health professional [3].

Therapeutic adherence is a complex process that is made up of a personal component represented by the patient, where their attitudes toward their disease and the positions they take on it are concentrated, as well as a relational component involving the health professional and the health structure that surrounds them. All these components work synergistically toward a common goal to benefit the patient’s health [4].

Adherence to ART is a critical component to achieve viral suppression in people living with HIV as well as to increase the overall quality of life [5]. Its study is important because in Mexico there has been a sustained prevalence in recent years, and it is necessary to improve the tools available to optimize treatment success [6].

There are various methods for measuring adherence in a patient; however, there is no gold standard for this purpose. Due to this, its measurement will depend on the characteristics of the population studied; in addition, the method should have basic psychometric standards of acceptable validity and reliability [7]. These methods are classified as direct, those that directly quantify the drug and its metabolites in blood or any other fluid or tissue, but they are costly and impractical for routine implementation, and indirect, that is they base their measurement on pill counts and self-reports, among others [8].

An indirect tool that has been used by several authors is the Simplified Medication Adherence Questionnaire (SMAQ). This questionnaire has been used in studies to evaluate adherence to ART in people living with HIV, and its six-question structure makes it practical in clinical contexts where a rapid evaluation is required [9,10]. This scale was introduced in 2002 by Knobel et al [9]. They designed the scale intending to create a questionnaire to identify nonadherent patients and found that this instrument had a sensitivity of 72%, a specificity of 91%, and a likelihood ratio of 7.94, as well as a Cronbach α of 0.75 [9].

The SMAQ questionnaire has also been validated in different pathologies, medical conditions, and chronic diseases, such as hypertension, diabetes, and tuberculosis. These validations have shown consistent results, supporting the usefulness and validity of the questionnaire in different health care settings [9,11]. The instrument was originally developed for the Spanish population in 1999, but the current social and pharmacological context differs for the population we are now studying. It is important to evaluate the validity and reliability of the instrument when used in contexts different from its original development, to ensure the quality of the collected information. This is because the metric quality of a self-report questionnaire must be explored in the context where it will be applied. Otherwise, the psychometric properties would be compromised, leading to negative consequences for the evaluation [12,13].

This questionnaire is not yet validated in Latin people, thus, its utility in our population is unknown. There is only a pilot study with 10 participants that used the SMAQ in Peruvian people living with HIV; where item comprehension was evaluated, as cultural applicability and social acceptance [14]. To our knowledge, no studies have been conducted to validate this instrument in the Mexican population. This study aimed to evaluate the reliability and validity of the SMAQ in people living with HIV/AIDS attending a Mexican national hospital.

## Methods

### Study Design

This study is a cross-sectional analytical design study concerning people living with HIV/AIDS receiving ART at the Civil Hospital of Guadalajara “Fray Antonio Alcalde.”

### Selection of Participants

Men older than 18 years of age receiving ART for at least three months before their inclusion in this study were included. Every participant gave his informed consent before participating in the research. On the other hand, those persons diagnosed with serious mental illnesses that may affect their ability to understand or answer the questionnaire were excluded, as well as those with cognitive or communication difficulties that may hinder their participation in the evaluation. Additionally considered as an exclusion criterion is not having performed the test to detect viral load within the period established for this study.

### Sample Size Calculation

To determine the sample size, we used a calculator that used a structural equation model approach, in which we anticipated an effect size (factor loading) of 0.5, which would reflect a significant contribution of the latent construct to the observed variables (items), ensuring sufficient construct reliability, a power of 80%, one latent variable, five observable variables, and a probability level of 0.05, resulting in a minimum sample of 100 participants, to detect the specified effect given the structural complexity of this model [15,16].

### Variables

The variables considered for this study were age in years, schooling (considered as the last completed grade of studies); municipality of residence (with the category “other” for those participants who indicated not being from a nearby municipality); marital status; employment (was considered positive when they would indicate having an economically remunerated activity); the use of tobacco, alcohol, and illicit drugs (it was considered positive when patients indicated to use in the last 30 days on more than two occasions); the number of pills (including those belonging to a treatment other than ART); time living with HIV and time on ART (which were calculated considering from the date of HIV diagnosis and the start of ART until the date of enrollment in this study); and finally, clinical stage was classified according to the Center of Disease Control classification [17].

### Instrument

The questionnaire consists of 6 questions: the first 4 can be answered with “yes” or “no,” while the last 2 require numerical responses: “1. Do you ever forget to take your medication?” “2. Do you always take your medication at the prescribed time?” “3. Do you ever stop taking the drugs if you feel unwell?” “4. Did you forget to take the medication over the weekend?” “5. In the last week, how many times did you miss a dose?” and “6. In the last 3 months, how many full days did you miss taking the medication?” The six questions assess three components of ART adherence: (1) the intentional (question 3), (2) the unintentional (questions 1 and 2), and (3) frequency or quantity (questions 4, 5, and 6). The patient is classified as nonadherent if they answer any in a “nonadherence sense” and if they report missing more than two doses in the last week or reports not having taken more than 2 full days of medication in the last three months [10].

### Statistical Analysis

For descriptive analysis, normality tests were performed as necessary. Quantitative variables were described with means or medians, and qualitative variables with frequencies and percentages.

The evidence of validity based on internal structure. was assessed by confirmatory factor analysis using the weighted least squares method with adjusted mean and variance [18]. Model fit was assessed using recognized indices, such as comparative fit index (CFI>0.90) [19], root-mean-square error of approximation (RMSEA<0.08) [20], and weight root-mean-square residual (WRMR<1) [21].

To determine reliability, we estimated the internal consistency coefficients according to Ponterotto and Charter [22]. Scores Cronbach α>0.70 were considered as reliable scores [22]. In addition, we investigated validity by examining the relationship between adherence and undetectable plasmatic viral load using the Pearson correlation coefficient [23].

Finally, Fisher exact tests were used to calculate the odds ratios (OR) to evaluate the association between having an undetectable (patient in control) versus detectable (patient not in control) plasmatic viral load and the scale classification of adherent versus nonadherent (based on the concept that a patient with an undetectable viral load was an adherent patient) [5].

The descriptive and correlational analyses were carried out in SPSS (version 24; IBM Corp) software, while the factor analysis was performed with Mplus (version 7; Muthén & Muthén) software [19]. Finally, the analysis of ORs was performed in Epi Info (Centers for Disease Control and Prevention) software in the Stat Cal module [24].

### Ethical Considerations

This research was approved by the Bioethics Committee of the Civil Hospital of Guadalajara (169/23), and it was carried out under the ethical standards for research on human participants. All participants signed an informed consent form that outlined the objectives of the research, the procedures to be followed, and the contact information for the research team. Any questions or concerns were addressed verbally prior to signing the consent form. The information collected was handled exclusively by the research team, and to protect participants' identities an alphanumeric code was assigned to each. Additionally, identifying data were excluded from the database used for analysis. Finally, no direct financial compensation was provided to participants for their involvement in the study.

## Results

### Characteristics of the Participants

A total of 299 patients were evaluated, 259 men and 40 women, and due to the ratio between groups, we decided to exclude the group of women from the analysis, to maintain the homogeneity and representativeness of the sample [25].

Of the 259 participants included, the mean age was 43 (SD 12) years with an average of 8.97 (SD 6.33) years in ART treatment. Considering schooling, the majority had high school (n=87, 34.6%) or middle school (n=57, 22%) education. The most common municipality of residence was Guadalajara (n=110, 42.5%) and the vast majority were single (n=214, 82.6%). Regarding substance use, 89.6% (n=232) had not consumed illicit substances in the last 30 days while 33.6% (n=87) had consumed alcohol and tobacco in the same period. The mean number of pills per day was 2 (SD 2) and the mean number of years living with HIV was 10 (SD 7.17) with an average CD4+ T lymphocyte count of 686.5 cells/μL (SD 353.88; Table 1).

**Table 1:** Sociodemographic characteristics of the sample (N=259).

		Values
Age (years), mean (SD)		43 (12)
**Schooling, n (%)**		
	Illiterate	2 (0.77)
	Can read and write	16 (6.2)
	Primary school	44 (17)
	Junior high school	57 (22)
	High school	87 (34.6)
	University	50 (19.3)
	Postgraduate	3 (1.2)
**Municipality of residence, n (%)**		
	Guadalajara	110 (42.5)
	Zapopan	32 (12.4)
	Tlaquepaque	24 (9.3)
	Tonalá	16 (6.2)
	Zapotlanejo	2 (0.8)
	Tepatitlán	1 (0.4)
	La Barca	1 (0.4)
	Arandas	2 (0.8)
	Zapotlán el Grande	2 (0.8)
	Other	69 (26.7)
**Marital status, n (%)**		
	Single	214 (82.6)
	Married	16 (6.2)
	Common law marriage	22 (8.5)
	Widower	2 (0.8)
**Regular employment, n (%)**		
	No	37 (14.3)
	Yes	222 (85.7)
**Used any illicit substance within the last 30 days, n (%)**		
	No	232 (89.6)
	Yes	27 (10.4)
**Consumed alcohol in the last 30 days, n (%)**		
	No	172 (66.4)
	Yes	87 (31.3)
**Used tobacco in the last 30 days**		
	No, n (%)	171 (66)
	Yes, n (%)	87 (33.6)
Pills taken per day, mean (SD)		2 (2)
Years in ART^a^, mean (SD)		8.97 (6.33)
Years living with HIV, mean (SD)		10.08 (7.17)
CD4+ T lymphocyte count, mean (SD)		686.47 (353.88)
**Clinical stage according to the CDC^b^, n (%)**		
	A1	122 (47.1)
	A2	20 (7.7)
	A3	4 (1.4)
	B1	16 (6.2)
	B2	4 (1.5)
	B3	4 (1.5)
	C1	37 (14.3)
	C2	32 (12.4)
	C3	20 (7.7)

^a^ART: antiretroviral therapy.

^b^CDC: Centers for Disease Control and Prevention.

### Responses From the Participants

Regarding the responses to the questionnaire, 66.6% (n=172) of the respondents reported not forgetting their medication, while 33.4% (n=87) admitted to having forgotten it at least once. On the second question, 80.3% (n=198) stated that they took their medication at the indicated time and 19.7% (n=61) admitted not following this pattern. Regarding whether they stopped taking the drugs if they felt unwell, the great majority (n=242, 92.2%) stated that they did not do so but 7.8% (n=17) admitted having done so at some time.

The majority reported not having forgotten to take their medication over the weekend (n=250, 96.6%). In the last week, the majority (n=242, 93.8%) reported missing only one dose, while 6.2% admitted missing a dose once or twice.

On the other hand, of the responses to question 6, a total of 66.5% (n=174) indicated not having missed taking medication on any full day, while 30.6% (n=174) acknowledged having experienced some degree of medication interruption.

Finally, according to the scores of the questionnaire, 66.6% (n=174) of participants reported being adherent while 33.4% (n=88) reported being nonadherent (Table 2).

**Table 2:** Distribution of responses to the Simplified Medication Adherence Questionnaire (N=259).

Questions			Participants, n (%)
**1. Do you ever forget to take your medication?**			
	No	172 (66.6)	
	Yes	87 (33.4)	
**2. Do you always take your medication at the prescribed time?**			
	No	61 (19.7)	
	Yes	198 (80.3)	
**3. Do you ever stop taking the drugs if you feel unwell?**			
	No	242 (92.2)	
	Yes	17 (7.8)	
**4. Did you forget to take your medication over the weekend?**			
	No	250 (96.6)	
	Yes	9 (3.4)	
**5. In the last week, how many times did you not take a dose?**			
	1	242 (93.8)	
	2	16 (5.9)	
	3	1 (0.3)	
**6. In the last 3 months, how many full days did you not take your medication?**			
	0	174 (66.5)	
	1	51 (20.6)	
	2	17 (5.9)	
	3	7 (3.1)	
	>4	10 (1)	
**Outcome on adherence**			
	Adherent	174 (66.6)	
	Nonadherent	88 (33.4)	

### Evidence of Validity Based on Internal Structure

The unidimensional model obtained favorable fit indices (CFI=1; RMSEA=0, 90% CI 0-0.085; WRMR=0.072), as well as factor loadings around what was expected, except for item 2 (Do you always take your medication at the prescribed time? Table 3). After performing a second analysis without item 2, the results are adequate both at the level of fit indices (CFI=1; RMSEA=0, 90% CI 0-0.103; WRMR=0.047) and factor loadings (>0.50; Table 3).

Regarding reliability, the magnitudes were acceptable for both scores (Cronbach α=0.702) and construct (ω=0.718).

Finally, the correlation of adherence with viral load was statistically significant (*r*=0.128; *P*=.04), but not with recent CD4 T lymphocyte counts (*r*=0.015; *P*=.81).

On the relationship between undetectable viral load and adherence, those patients classified as adherent by the scale were 3 times more likely to be undetectable compared to those classified as nonadherent (OR 3.31, 95% CI 1.13-9.64, *P*=.04; Table 4).

**Table 3:** Factor loadings of the Simplified Medication Adherence Questionnaire scale.

Questions	First analysis	Second analysis
1. Do you ever forget to take your medication?	0.815	0.854
2. Do you always take your medication at the prescribed time?	–0.358	—^a^
3. Do you ever stop taking drugs if you feel sick or drink alcohol?	0.612	0.526
4. Did you forget to take your medication over the weekend?	0.383	0.374
5. In the last week, how many times did you not take a dose?	0.677	0.701

^a^Not applicable.

**Table 4:** Relationship between Simplified Medication Adherence Questionnaire adherence classification and viral load.

	Adherent	Nonadherent	OR^a^ (95% CI)	*P* value
Undetectable viral load^b^	168	76	3.31 (1.13-9.64)	.04
Detectable viral load^c^	6	9	—^d^	—

^a^OR: odds ratio.

^b^Plasma viral load of ≤40 copies/mL.

^c^Plasma viral load >40 copies/mL.

^d^Not applicable.

## Discussion

### Principal Findings

Adherence is vital to achieve viral suppression and increase the quality of life of people living with HIV; however, its measurement represents a major challenge [26]. Although direct methods exist to measure adherence (eg, plasma drug concentration), they are costly and impractical for routine implementation in clinical settings with limited resources such as ours [8], thus, indirect measurement methods based on self-reporting may have a good performance and could be used routinely.

This study aimed to evaluate the reliability and validity of the SMAQ in people living with HIV/AIDS attending a public hospital in the western region of Mexico.

As for the factorial structure, 4 of the 5 original items were retained, and the one that was eliminated obtained the lowest factorial loading, and its response was oriented in the opposite direction to the other items, which reinforces the argument that this type of item usually presents methodological problems. This new four-item version obtained adequate fit and reliability indices (CFI=1; RMSEA=0, 90% CI 0-0.103; WRMR=0.047) [27].

The validity of its relationship with other variables was analyzed using an association with viral suppression (or undetectable viral load), finding a threefold greater probability of achieving viral suppression when classified as adherent (OR 3.31, 95% CI 1.13-9.64, *P*=.04).

The use of the SMAQ in the framework of explanatory studies could provide greater insight into the factors associated with adherence, providing evidence for the design of appropriate interventions to improve adherence in this patient population [28]. This, in turn, would strengthen the capacity of health services to enhance and promote quality care, ultimately yielding a positive long-term impact on people living with HIV.

Concerning previous studies, the study by Agala et al [10] also found adequate factor loadings, except for one item, like this work. However, there is no agreement on the eliminated item, which could be attributed to differences in the populations studied because the analysis of our study was only in men and theirs in women. This highlights the existing differences in adherence behaviors between these groups [10].

This situation underscores the need to consider sociodemographic and cultural characteristics when analyzing the evidence and validity of self-report tools such as the SMAQ.

### Limitations

Regarding its limitations, it is important to recognize that, despite its simplicity, it may be subject to self-report bias [11]. Similarly, only men were considered due to the proportion that was recruited from both groups and to maintain statistical homogeneity [29]. This limitation does not allow for the opportunity to explore differences that could influence adherence between the two groups.

It is concluded that the SMAQ presents favorable evidence of validity per internal structure and association with viral suppression, as well as acceptable levels of reliability.

In future studies, we recommend that the SMAQ be analyzed at a psychometric level in the group of women to explore possible differences by gender. In addition, this tool will allow studies focused on adherence and its determinants to be carried out with a greater degree of precision.

### Conclusions

The use of the SMAQ provides a valuable tool for assessing adherence among the Mexican population living with HIV. This evaluation is critical for enhancing health outcomes and optimizing therapeutic interventions. Additionally, the SMAQ helps to strengthen public health programs by furnishing reliable data on treatment adherence, which can inform the development of targeted strategies to support patient engagement and medication management.

## Abbreviations

ART: antiretroviral treatment
CFI: comparative fit index
OR: odds ratio
RMSEA: root-mean-square error of approximation
SMAQ: Simplified Medication Adherence Questionnaire
WRMR: weight root-mean-square residual
